# The family-specific α4-helix of the kinesin-13, MCAK, is critical to microtubule end recognition

**DOI:** 10.1098/rsob.160223

**Published:** 2016-10-12

**Authors:** Jennifer T. Patel, Hannah R. Belsham, Alexandra J. Rathbone, Bill Wickstead, Christopher Gell, Claire T. Friel

**Affiliations:** School of Life Sciences, University of Nottingham, Medical School, QMC, Nottingham NG7 2UH, UK

**Keywords:** MCAK, kinesin-13, microtubule, depolymerization, ATP turnover, microtubule end recognition

## Abstract

Kinesins that influence the dynamics of microtubule growth and shrinkage require the ability to distinguish between the microtubule end and the microtubule lattice. The microtubule depolymerizing kinesin MCAK has been shown to specifically recognize the microtubule end. This ability is key to the action of MCAK in regulating microtubule dynamics. We show that the α4-helix of the motor domain is crucial to microtubule end recognition. Mutation of the residues K524, E525 and R528, which are located in the C-terminal half of the α4-helix, specifically disrupts the ability of MCAK to recognize the microtubule end. Mutation of these residues, which are conserved in the kinesin-13 family and discriminate members of this family from translocating kinesins, impairs the ability of MCAK to discriminate between the microtubule lattice and the microtubule end.

## 1. Introduction

Kinesins are a superfamily of proteins characterized by a common highly conserved motor domain [1,2]. Kinesin activity can be broadly divided into two classes: (i) translocating activity, which is moving directionally along the microtubule lattice; and (ii) microtubule-regulating activity, which is altering microtubule growth and shrinkage dynamics, with some families displaying activity from each class. All kinesins which regulate microtubule dynamics studied to date have the ability to distinguish between the microtubule end and the microtubule lattice. For example, the kinesin-5 (Eg5), which enhances microtubule polymerization, pauses at the microtubule plus end [3]. The kinesin-7 (CENP-E) and the non-motile kinesin (NOD), are both suggested to promote polymerization and have been shown to localize preferentially to microtubule ends [4,5]. The kinesin-8 (Kip3), which depolymerizes microtubules, resides on the microtubule plus end longer than its stepping time on the microtubule lattice [6]. Thus, the ability to distinguish microtubule lattice from microtubule end appears to be a common theme among microtubule-regulating kinesins.

The molecular mechanism underlying specific recognition of the microtubule end by a kinesin is not clear. Here, we focus on the microtubule depolymerizing kinesin-13 (MCAK), which has been shown to discriminate microtubule end from lattice [7–9]. Depolymerizing kinesins play a crucial role in the control of microtubule length distributions in the cell [10,11]. The ATP turnover cycle of MCAK is atypical, with the rate-limiting step in the absence of tubulin being ATP cleavage rather than ADP dissociation [8]. Tubulin in any form (unpolymerized, microtubule lattice or microtubule end) accelerates ATP cleavage such that ADP dissociation becomes rate-limiting. While the ATPase activity of MCAK is partially stimulated by any form of tubulin, microtubule ends maximally stimulate the ATPase by accelerating ADP dissociation (electronic supplementary material, figure S1). Only microtubule ends have the ability to accelerate ADP dissociation from the MCAK motor domain.

Here, we show that three residues (K524, E525 and R528), which are located in the C-terminal half of the α4-helix, play a crucial role in the ability of MCAK to distinguish between the microtubule lattice and the microtubule end. Mutation of each of these residues to alanine not only reduces the residence time of MCAK at the microtubule end, but also impairs the ability of the microtubule end to accelerate ADP dissociation from the MCAK motor domain.

## 2. Results

### 2.1. Mutations in the α4-helix impair depolymerization activity and microtubule-stimulated ATPase

To better understand what differentiates translocating kinesins from microtubule-regulating kinesins, we made mutations to the MCAK motor domain based on a comparative protein sequence alignment of the motor domains of the kinesin-1 and kinesin-13 families (electronic supplementary material, figure S2). We screened these mutants for depolymerization activity (electronic supplementary material, figure S3). The most potent mutations were in sequence motifs previously shown to impair microtubule depolymerization activity: Loop 2 and α4-helix [12,13]. Mutations in Loop 8 and Loop 11 also had a significant effect on microtubule depolymerization. However, many positions shown by comparative alignment to discriminate between the kinesin-13 and kinesin-1 families had little effect on the depolymerization activity of human MCAK.

**Figure 1.**
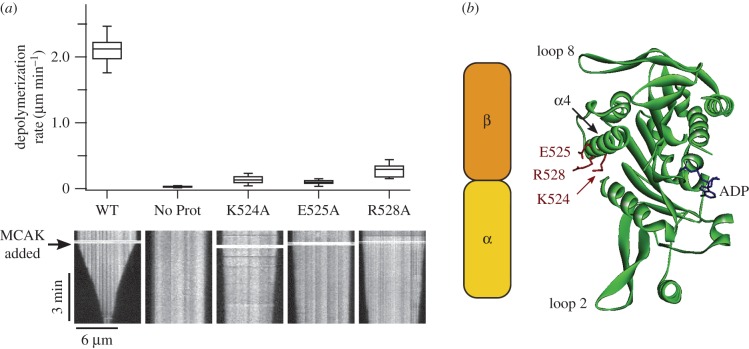
(*a*) Microtubule depolymerization rates of WT-MCAK and three variants: K524A, E525A and R528A. Box plots show the distribution of depolymerization rates for individual microtubules upon the addition of 40 nM WT-MCAK or MCAK variant and in the absence of MCAK (No Prot). A representative kymograph is shown for each condition. Addition of MCAK is indicated by the black arrow. (*b*) Crystal structure of the motor domain of human MCAK (PDB ID: 2HEH). The locations of the three residues of interest in the α4-helix are marked. A schematic of an α/β-tubulin heterodimer is included to show the orientation of tubulin when in complex with the MCAK motor domain [14]. The α4-helix binds in the intradimer groove between the α- and β-subunits of the tubulin heterodimer.

We chose to study in detail three mutants in the α4-helix, K524A, E525A and R528A, each of which reduced depolymerization activity by over 80% relative to wild-type MCAK (electronic supplementary material, figure S3). These three residues are highly conserved across the kinesin-13 family and their mutation to alanine has previously been shown to impair both the depolymerization activity and the microtubule-stimulated ATPase of a motor-domain-only construct of *Plasmodium falciparum* MCAK [13]. In agreement with this, depolymerization rates of individual microtubules for K524A, E525A and R528A were significantly reduced relative to WT-MCAK: 0.13 ± 0.05 µm min^−1^, 0.10 ± 0.03 µm min^−1^, 0.29 ± 0.09 µm min^−1^ and 2.12 ± 0.17 µm min^−1^, respectively (figure 1*a* and table 1). We need to understand the mechanism by which mutation of these residues, which are located on the tubulin binding face of the α4-helix (figure 1*b*), affects the activity of MCAK to better understand the function of the α4-helix in the process of microtubule depolymerization.

**Table 1.** Compiled values for the depolymerization rate ATPase activities, microtubule-end residence time and mantADP dissociation for WT-MCAK and the mutants K524A, E525A and R528A.

MCAK variants		WT	K524A	E525A	R528A
		(μm min^−1^) (mean ± s.d.)			
depolymerization rate		2.12 ± 0.17 (*n* = 18)	0.13 ± 0.05 (*n* = 16)	0.10 ± 0.03 (*n* = 16)	0.29 ± 0.09 (*n* = 14)
ATPase activity		ATPase (s^−1^) (mean ± s.e.m.)			
solution (basal)		2.11 ± 0.31 × 10^−3^ (*n* = 4)	2.15 ± 0.31 × 10^−3^ (*n* = 3)	2.21 ± 0.04 × 10^−3^ (*n* = 3)	3.68 ± 0.02 × 10^−3^ (*n* = 3)
tubulin-stimulated		0.204 ± 0.041 (*n* = 7)	0.184 ± 0.035 (*n* = 4)	0.195 ± 0.043 (*n* = 4)	0.205 ± 0.054 (*n* = 4)
microtubule-stimulated		3.16 ± 0.43 (*n* = 6)	0.72 ± 0.23 (*n* = 3)	0.16 ± 0.05 (*n* = 3)	0.66 ± 0.15 (*n* = 3)
		(s) (mean ± s.e.m.)			
microtubule-end residence time		2.03 ± 0.13 (*n* = 238)	0.59 ± 0.02 (*n* = 260)	0.59 ± 0.02 (*n* = 229)	0.65 ± 0.02 (*n* = 264)
mantADP dissociation		rate constant (s^−1^) (mean ± s.e.m.)			
solution		0.125 ± 0.003 (*n* = 5)	0.128 ± 0.001 (*n* = 4)	0.125 ± 0.003 (*n* = 4)	0.133 ± 0.006 (*n* = 4)
tubulin-stimulated		0.132 ± 0.006 (*n* = 3)	0.123 ± 0.001 (*n* = 3)	0.118 ± 0.003 (*n* = 3)	0.120 ± 0.002 (*n* = 3)
microtubule-stimulated^a^	first phase	2.84 ± 0.26 (*n* = 4)	0.75 ± 0.08 (*n* = 3)	n.a.	1.07 ± 0.12 (*n* = 4)
	second phase	0.266 ± 0.037 (*n* = 4)	0.172 ± 0.003 (*n* = 3)	0.178 ± 0.003 (*n* = 3)	0.207 ± 0.005 (*n* = 4)

^a^For microtubule-stimulated mantADP dissociation the data for WT-MCAK, K524A and R528A were fitted to a double exponential function. Therefore, two rate constants were obtained, labelled first and second phases. The data for E525A fit well to a single exponential and so only one rate constant was obtained for this mutant.

**Figure 2.**
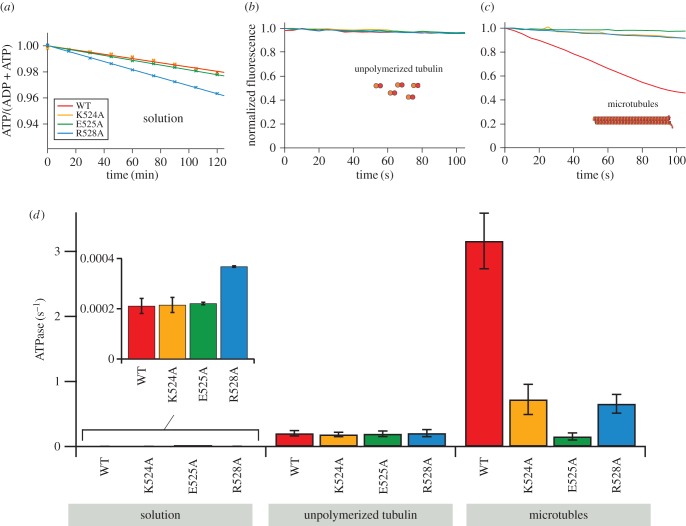
Microtubule-stimulated ATPase is reduced by mutations to the α4-helix. (*a*) Change in fraction of ATP upon addition of WT-MCAK or MCAK variants K524A, E525A or R528A in solution (the absence of tubulin and microtubules). (*b,c*) Change in fluorescence intensity of NADH, which reports on production of ADP, upon addition of WT-MCAK or the MCAK variants K524A, E525A or R528A in the presence of (*b*) 10 µM GDP.tubulin or (*c*) 10 µM GMPCPP-stabilized microtubules. (*d*) Bar chart showing ATPase (mean±s.e.m.) for WT-MCAK and K524A, E525A and R528A in solution, in the presence of unpolymerized tubulin and in the presence of microtubules. Inset: ATPase in solution shown on a different scale.

To understand the mechanism by which the mutations K524A, E525A and R528A attenuate microtubule depolymerization activity, we measured their ATPase activity (product produced per motor domain per second) in solution (basal ATPase), in the presence of unpolymerized tubulin (tubulin-stimulated ATPase) and in the presence of microtubules (microtubule-stimulated ATPase; figure 2). The basal ATPase for each mutant was measured by a discontinuous assay in which the production of ADP was followed using HPLC (figure 2*a*). We use this assay as it is sufficiently sensitive to detect the low level of ATPase activity of MCAK in solution (approx. 2 × 10^−3^ s^−1^). We then measured the ATPase for each mutant in the presence of unpolymerized tubulin (figure 2*b*) and of microtubules (figure 2*c*), using an enzyme-linked assay to follow the production of ADP. None of the mutations resulted in a major change in basal ATPase activity or in tubulin-stimulated ATPase activity (figure 2*d* and table 1). By contrast, the microtubule-stimulated ATPase for each mutant was significantly reduced (figure 2*d*). The microtubule-stimulated ATPases for K524A and R528A were 4.5-fold and 4.8-fold lower, respectively, than the microtubule-stimulated ATPases for WT-MCAK: 0.72 ± 0.23 s^−1^, 0.66 ± 0.15 s^−1^ and 3.16 ± 0.43 s^−1^ (figure 2*d* and table 1). The mutation E525A had a greater effect, reducing the microtubule-stimulated ATPase by 20-fold to 0.16 ± 0.05 s^−1^.

Thus, each of the mutants turns over ATP in solution and in the presence of unpolymerized tubulin at the same or similar rate to WT-MCAK, indicating that these mutations have altered neither the structure of the nucleotide-binding site of the MCAK motor domain nor its ability to interact with unpolymerized tubulin. By contrast, each mutation significantly reduced the microtubule-stimulated ATPase relative to WT-MCAK, indicating that their reduced depolymerization activity results from disruption of an interaction between MCAK and microtubules which is not formed with unpolymerized tubulin.

### 2.2. Mutations in the α4-helix do not affect interaction with the microtubule lattice

**Figure 3.**
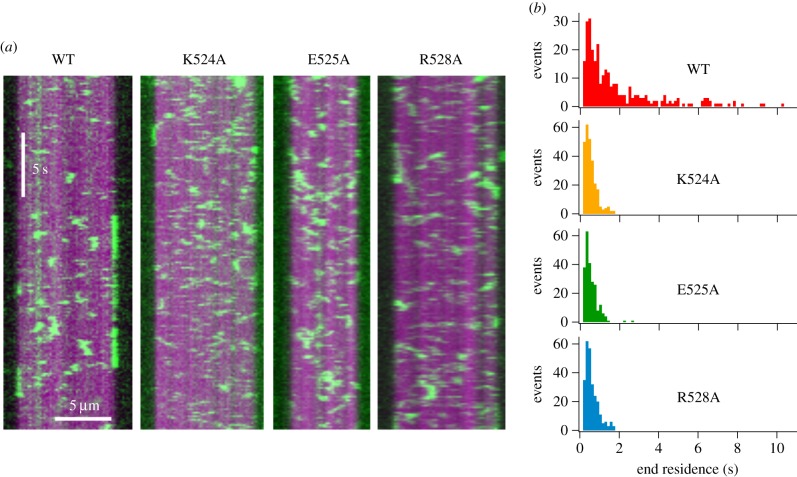
Mutations to the α4-helix of MCAK reduce the microtubule-end residence time. (*a*) Representative kymographs showing the interaction of WT-MCAK-GFP and GFP-tagged mutants (green) with GMPCPP-stabilized microtubules (magenta). (*b*) Histograms showing the number of microtubule-end interaction events of a particular residence time for WT-MCAK (*n* = 276), K524A (*n* = 260), E525A (*n* = 229) and R528A (*n* = 264).

The simplest explanation for the pattern of ATPase activities observed for the mutants K524A, E525A and R528A is that they have abolished the ability of MCAK to interact with the microtubule. MCAK has two modes of microtubule interaction: (i) a diffusive interaction with the lattice [15,16], and (ii) a tightly bound interaction with the microtubule end [7,9,17]. These mutations may disrupt one or both of these modes of interaction. To distinguish between the diffusive interaction with the microtubule lattice and the tightly bound complex at the microtubule end, we observed GFP-tagged WT-MCAK and mutants at single-molecule concentrations using TIRF microscopy. Numerous lattice interaction events were observed for WT-MCAK and each of the mutants (figure 3*a*). There was no significant difference in the duration of lattice interaction events between WT-MCAK and the mutants (electronic supplementary material, figure S4): neither the mean lattice residence time nor lattice dissociation constant (*k*_off_) was affected by these mutations (electronic supplementary material, table S1). Furthermore, there was no significant effect on the microtubule association rate (*k*_on_) (electronic supplementary material, table S1). Taken together these data indicate that the mutations K524A, E525A and R528A have not changed the affinity of MCAK for the microtubule lattice. These data are contrary to previous work which showed that kinesin-13 constructs containing one or more of the same mutations did not bind microtubules [13,14]. This difference is probably due to monomeric motor-domain-only constructs previously used compared with full-length dimeric MCAK used here.

### 2.3. Mutations in the α4-helix reduce residence time at the microtubule end

As the interaction of MCAK with the microtubule lattice was not altered by these mutations, we then focused on the interaction between MCAK and the microtubule end. For both WT-MCAK and the mutants, many end interaction events were observed (figure 3). However, the duration of these events was dramatically different for the mutants relative to WT-MCAK. Both WT-MCAK and the mutants had many end interaction events lasting less than 2 s. However, in addition to these short interactions WT-MCAK also had many longer interactions with the microtubule end. For WT-MCAK, 31.5% of microtubule end interaction events were longer than 2 s (figure 3*b*). By contrast, for K524A, E525A and R528A, only 0%, 0.9% and 0%, respectively, of observed microtubule end interaction events were longer than 2 s (figure 3*b*).

The mean end residence times for WT-MCAK, K524A, E525A and R528A were 2.03 ± 0.13 s, 0.59 ± 0.02 s, 0.59 ± 0.02 s and 0.65 ± 0.02 s, respectively (table 1). Thus, each of these mutations reduced the mean end residence time for MCAK more than threefold. These mutations have caused MCAK to lose the long end residence events observed for the wild-type (figure 3*b*). The microtubule end residence times for the mutants are not significantly different to the lattice residence times for both WT-MCAK and mutants. These data suggest that the mutations have impaired and possibly abolished the ability of MCAK to discriminate between the microtubule lattice and the microtubule end.

### 2.4. Microtubule-end-accelerated ADP dissociation is slowed or abolished by mutations in the α4-helix

**Figure 4.**
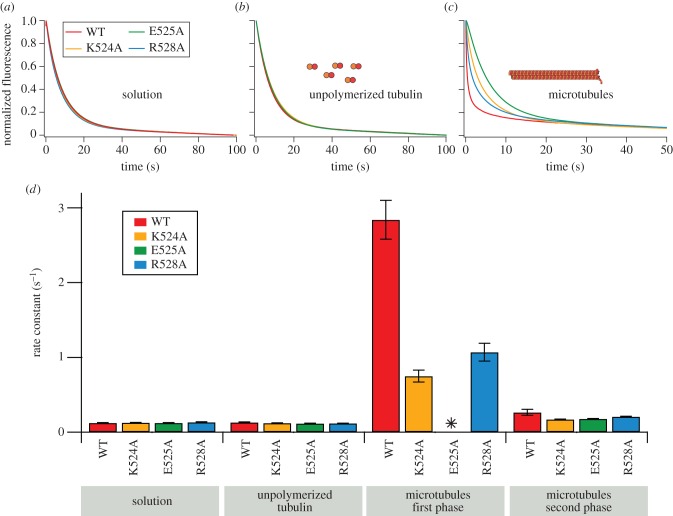
Mutations to the α4-helix reduce the microtubule-end-stimulated rate constant for mantADP dissociation. (*a*–*c*) Representative fluorescence traces for the dissociation of mantADP from WT-MCAK and the variants K524A, E525A and R528A: (*a*) in solution (the absence of tubulin and microtubules), (*b*) in the presence of 10 µM unpolymerized GDP.tubulin and (*c*) in the presence of 5.7 µM double-cycled GMPCPP microtubules. The fits to these data are shown in electronic supplementary material, figure S5. (*d*) Bar chart showing rate constants for the dissociation of mantADP (mean±s.e.m.) for WT-MCAK and K524A, E525A and R528A in solution, in the presence of unpolymerized tubulin and in the presence of microtubules. The decrease in fluorescence upon dissociation of mantADP from WT-MCAK, K524A and R528A in the presence of microtubules is fitted to a double exponential function; therefore, two rate constants are obtained (first and second phases). Asterisk denotes fluorescence decrease for E525A is fitted to a single exponential function; therefore, a single rate constant is obtained.

The microtubule end maximally activates the ATPase activity of MCAK by accelerating ADP dissociation [8]. Only the microtubule end accelerates ADP dissociation from MCAK; therefore, tubulin in other forms only partially activates the ATPase (electronic supplementary material, figure S1). Our data indicate that the mutations K524A, E525A and R528A disrupt the ability of MCAK to discriminate between the microtubule end and the lattice. Thus, the ability of microtubule ends to accelerate ADP dissociation should also be impaired by these mutations. To test this, we measured the kinetics of ADP dissociation by preloading the nucleotide-binding site with a fluorescent ADP analogue (mantADP) and using rapid mixing to observe the fluorescence decrease associated with its dissociation from the motor domain (figure 4*a*–*c*) [18,19]. The rate constant for mantADP dissociation from MCAK in solution and in the presence of unpolymerized tubulin is unchanged by the mutations (figure 4*d* and table 1). By contrast, the kinetics of microtubule-stimulated dissociation of mantADP are significantly altered relative to WT-MCAK (figure 4*d* and table 1).

The decrease in fluorescence observed upon dissociation of mantADP from WT-MCAK in the presence of microtubules is well described by a double exponential function (figure 4*c*; electronic supplementary material, figure S5). The first (faster) phase results from microtubule end-stimulated dissociation of mantADP. The second (slower) phase reflects mantADP dissociation from molecules that fail to reach a microtubule end. These could be molecules from which mantADP dissociates when in solution, in contact with unpolymerized tubulin or in contact with the microtubule lattice [8]. In support of this interpretation, the rate constant of the second phase agrees with that for mantADP dissociation from MCAK in solution and in the presence of unpolymerized tubulin (figure 4*d* and table 1).

The decrease in fluorescence upon mantADP dissociation from the mutants K524A and R528A in the presence of microtubules remained well described by a double exponential function (figure 4*c*; electronic supplementary material, figure S5). However, while the rate constant for the second phase was unaffected, the rate constant for the first phase was significantly reduced: 0.75 ± 0.08 s^−1^, 1.07 ± 0.12 s^−1^ and 2.84 ± 0.26 s^−1^ for K524A, R528A and WT, respectively (figure 4*d* and table 1). Thus, the microtubule-end-stimulated mantADP dissociation constant is reduced 3.8-fold and 2.7-fold by K524A and R528A, respectively.

The mutation E525A had the greatest effect on microtubule-end-stimulated ADP dissociation. The fluorescence decrease observed upon dissociation of mantADP from E525A in the presence of microtubules did not require fitting to a double exponential function but was well described by a single exponential (figure 4*c*; electronic supplementary material, figure S5). The first (faster) phase, attributed to microtubule-end-stimulated mantADP dissociation, was not observed. The rate constant determined for the single exponential was 0.178 ± 0.003 s^−1^, which is consistent with the rate constant for the second phase of the double exponential traces observed for WT, K524A and R528A (figure 4*d* and table 1). These data suggest that the mutation E525A has affected the interaction of MCAK with the microtubule end such that the microtubule end does not accelerate ADP dissociation. Rather, for E525A, microtubule-end-stimulated mantADP dissociation occurs with the same rate constant as lattice tubulin or unpolymerized tubulin-stimulated mantADP dissociation. Therefore, ADP dissociation at the microtubule end is not distinguishable from ADP dissociation on the microtubule lattice. Thus, the fluorescence decrease upon mantADP dissociation for this mutant is described by a single exponential. These data indicate that mutation of E525 eliminates the ability of MCAK to discriminate between microtubule lattice and end.

## 3. Discussion

### 3.1. The α4-helix is critical for MCAK to distinguish between the microtubule lattice and end

Each of the mutations to the α4-helix of the MCAK motor domain significantly reduces the microtubule-end residence time, while having no effect on the lattice residence time or the affinity of MCAK for the microtubule lattice. This contrasts with previous work in which kinesin-13 constructs containing one or more of the same mutations do not bind any region of the microtubule [13,14]. This difference is probably due to previous work using monomeric motor-domain-only constructs rather than full-length dimeric MCAK. These observations suggest that both motor domains of the MCAK dimer make a contribution to lattice affinity and/or that regions outside the motor domain are involved in the diffusive interaction with the lattice. Previous work has shown that regions outside the motor domain do play a role in regulating affinity for the microtubule [20–22]. The data presented here indicate that the motor domain (and in particular the α4-helix) is critical for MCAK to discriminate the microtubule end from the lattice. In terms of residence time, there is a clear distinction between the microtubule lattice and microtubule end for WT-MCAK, whereas for the mutants K524A, E525A and R528A there is no significant difference. Our data indicate that it is the duration, rather than the number, of end interaction events that is altered by these mutations. This suggests that the short (less than 2 s) interactions with the microtubule end are mainly not productive for removal of tubulin.

### 3.2. Microtubule-end recognition via the α4-helix promotes nucleotide exchange

Each of the mutations studied here significantly reduces the microtubule-end-stimulated rate constant for ADP dissociation. Indeed, E525A reduces this rate constant such that it becomes indistinguishable from lattice-stimulated ADP dissociation. These data indicate that the α4-helix is crucial to microtubule-end-stimulated nucleotide exchange. By diminishing the ability of MCAK to recognize the microtubule end, these mutations slow end-stimulated ADP dissociation and therefore slow the exchange of ADP for ATP promoted by the microtubule end. By reducing the probability of exchange of ADP for ATP the tightly bound ATP.MCAK complex formed at the microtubule end is disfavoured. This leads to the observed reduction in microtubule-end residence time and diminished depolymerization activity. Thus, our data indicate that these residues in the α4-helix are crucial to the interaction with the microtubule end which promotes ADP release from the nucleotide-binding site.

### 3.3. MCAK resides at the microtubule end for many rounds of ATP turnover

The lifetime of ATP.MCAK in the presence of microtubules is 0.1 s [8]. This suggests that, during the mean end residence time for WT-MCAK of 2 s (table 1) [15], a single MCAK molecule remains associated with the microtubule end for on average 20 cycles of ATP turnover. It remains to be determined how MCAK maintains this association through rounds of ATP turnover during which the motor domain will cycle between states of high and low affinity for tubulin. It is probable that, since MCAK is a dimer, the two motor domains are coordinated such that one motor domain has high affinity for tubulin when the other has low affinity.

### 3.4. Structural basis of microtubule-end recognition by MCAK

Available structures of kinesin-13 motor domains bound to tubulin [14] or docked onto the structure of the microtubule [12] shed light on why mutations in the α4-helix should disrupt MCAK's interaction with the microtubule end but not with unpolymerized tubulin or the microtubule lattice. A cryoEM structure of a *Drosophila* homologue of MCAK in complex with tubulin, in the form of protofilament rings, shows the α4-helix bound in the intradimer groove of a tubulin heterodimer that is deformed by the interaction with the kinesin-13 motor domain [14]. None of the residues equivalent to K524, E525 or R528 interact with tubulin in this structure. Our observation that mutation of these residues has no effect on the ATP turnover cycle of MCAK in the presence of unpolymerized tubulin but a large effect on the turnover cycle in the presence of microtubules suggests that the protofilament ring complex probably mimics the interaction of kinesin-13 with unpolymerized tubulin rather than the complex formed at the microtubule end.

A model of the mouse MCAK motor domain docked *in silico* onto a curved tubulin heterodimer, as is suggested to exist at the microtubule end, indicates that the α4-helix has the potential to fit well in the intradimer groove of curved α/β-tubulin [12]. This is not the case when MCAK is docked onto a straight tubulin dimer, as exists within the microtubule lattice. The residues equivalent to K524, E525 and R528 (K520, E521 and R524) form part of the binding interface in the complex of MCAK with curved tubulin in this model. That the α4-helix, and these residues in particular, form a better interface with curved tubulin than straight tubulin suggests a mechanism by which the α4-helix may allow MCAK to distinguish between the microtubule end and the lattice.

### 3.5. Structural basis of microtubule-end-stimulated ADP dissociation

Recently published structures of the kinesin-1 motor domain in complex with tubulin have highlighted the interaction of the α4-helix with tubulin as a major driver of conformational changes in the nucleotide-binding site [23,24]. Here, we show that the mutated residues, which are in the C-terminal half of the α4-helix, are not important for either microtubule or unpolymerized tubulin activation of the ATP cleavage step of ATP turnover by MCAK. However, these residues are vital for the activation of ADP dissociation by the microtubule end. This suggests that the C-terminal half of the α4-helix is not involved in driving the conformational change of the switch loops (L9 and L11), which renders the nucleotide-binding site competent to hydrolyse ATP, upon interaction with tubulin [24]. They are, however, vital to microtubule-end-stimulated release of ADP, suggesting they drive opening of the switch loops upon interaction with the microtubule end.

### 3.6. Implications for regulation of the activity of MCAK

The requirement to recognize the end of the microtubule to facilitate depolymerization appears to be exploited by regulatory proteins to alter the activity of MCAK. For example, the microtubule tip tracking protein EB3 increases the catastrophe-causing activity of MCAK by localizing it to the microtubule plus end via a direct interaction between EB3 and MCAK [25]. The increased activity of MCAK in the presence of EB3 is probably due to an effective higher concentration of MCAK at the microtubule end due to an increased end residence time. By contrast, the microtubule-stabilizing protein XMAP215 suppresses the microtubule catastrophe-inducing activity of the *Xenopus* MCAK orthologue, XKCM1 [26]. XMAP215 is a microtubule + TIP tracking protein which promotes microtubule polymerization. XMAP215 may suppress the activity of XKCM1 by occluding or eradicating the particular feature of the microtubule end recognized by kinesin-13s.

### 3.7. Molecular mechanism of microtubule-end recognition and depolymerization

**Figure 5.**
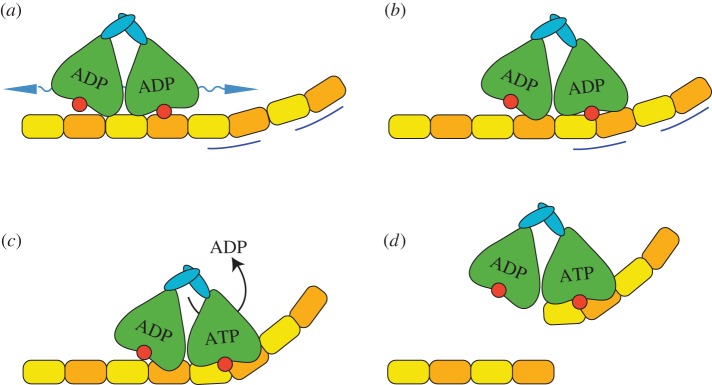
Possible mechanism of microtubule end recognition and depolymerization. (*a*) MCAK reaches the microtubule end principally by lattice diffusion in the ADP-bound state. (*b*) MCAK recognizes a structural feature found at or near the microtubule end (indicated by blue lines). The α4-helix (red circle) is critical to this recognition. (*c*) The interaction between MCAK and the microtubule end accelerates dissociation of ADP, thereby promoting nucleotide exchange. ATP.MCAK binds tightly to tubulin, forming a depolymerization-competent complex at the microtubule end. It is not currently known if and how the two motor domains of the MCAK dimer are coordinated during this process. (*d*) Binding of ATP.MCAK probably deforms the tubulin dimer, thereby promoting dissociation of tubulin.

MCAK reaches the microtubule end in an ADP-bound state primarily via lattice diffusion (figure 5*a*). MCAK can also directly bind the microtubule end; however, the larger number of lattice binding sites versus end binding sites suggests the majority of molecules reach the end via lattice diffusion [15,16]. Upon reaching the microtubule end, MCAK recognizes a structural feature associated with this region (figure 5*b*). Available evidence suggests that this feature is probably a particular degree of curvature or flexibility of tubulin found only at or near the microtubule end [12,27]. Another possibility is that MCAK recognizes the nucleotide state of tubulin at the microtubule end; however, MCAK can distinguish lattice from end in microtubules containing only one nucleotide type, suggesting that this is a less likely mechanism of end recognition. It is also possible that the absence of a longitudinal neighbour of the terminal tubulin is the feature by which MCAK distinguishes the microtubule end. Whatever the microtubule-end-specific feature, our data suggest that the α4-helix plays a crucial role in its recognition. Interaction of the motor domain with the microtubule end accelerates ADP dissociation, stimulating exchange of ADP for ATP (figure 5*c*). In the ATP-bound state, MCAK binds more tightly to tubulin, forming a depolymerization-competent complex at the end of the microtubule. This interaction probably promotes microtubule depolymerization by the deformation of the bound tubulin dimer (figure 5*d*). It remains to be determined whether deformation of tubulin dimers is sufficient to cause their dissociation from the microtubule or whether active severing at the interdimer interfaces is also required. It could be that structural features of the motor domain suggested to contact the interdimer interface, such as Loop 2 and Loop 8 [12,14], destabilize longitudinal interactions.

It is possible that structural elements of the kinesin-13 motor domain in addition to the α4-helix are required for microtubule-end recognition. However, it is clear from the work described here that the α4-helix is critical to enable discrimination of microtubule lattice from microtubule end. It will be interesting to discover whether this role of the α4-helix in microtubule-end recognition by kinesin-13s is shared by other microtubule-regulating kinesins.

## 4. Material and methods

### 4.1. Proteins

Full-length human MCAK-his6, MCAK-his6-EGFP and all variants of these proteins were expressed in Sf9 cells and purified using cation exchange and Ni-affinity chromatography [15]. MCAK concentrations are given as the concentration of monomer.

Porcine brain tubulin was isolated as described in [28].

### 4.2. Microtubules

Single-cycled, rhodamine-labelled, GMPCPP-stabilized microtubules were prepared by incubating 2 µM tubulin containing 25% rhodamine-labelled tubulin in BRB80 (80 mM PIPES pH 6.9, 1 mM MgCl_2_, 1 mM EGTA) supplemented with 1 mM GMPCPP for 2 h at 37°C. Microtubules were pelleted using a Beckman Airfuge Ultracentrifuge and resuspended in BRB80 buffer to be used directly.

Double-cycled, GMPCPP-stabilized microtubules were made as described in [29]*.* These microtubules were stored in liquid N_2_ and thawed at 37°C immediately prior to use.

The concentration stated for microtubules is the concentration of polymerized tubulin.

### 4.3. Microtubule depolymerization by fluorescence microscopy

Single-cycled, rhodamine-labelled, GMPCPP-stabilized microtubules were adhered to the surface of flow chambers prepared from treated coverslips as described in [15,29]. Images of a field of fluorescent microtubules were recorded on a DeltaVision Elite microscope (Applied Precision/GE Healthcare) equipped with a 100× objective (UPlanSApo/1.4NA oil, Olympus) and an Evolve 512 EMCCD camera (Photometrics). Experiments were performed at 25°C. In total, 40 nM MCAK or MCAK mutants were added in BRB20 pH 6.9, 75 mM KCl, 1 mM ATP, 0.05% Tween 20, 0.1 mg ml^−1^ BSA, 1% 2-mercaptoethanol, 40 mM glucose, 40 mg ml^−1^ glucose oxidase, 16 mg ml^−1^ catalase to the channel 1 min after acquisition had commenced. Depolymerization rates were determined from plots of the length of individual microtubules versus time, obtained by thresholding and particle analysis of images using Fiji [30].

### 4.4. ATPase activity

Assays were performed in BRB80 pH 6.9, 75 mM KCl, 2 mM MgATP, 1 mM DTT, 0.05% Tween at 25°C. Assays were initiated by the addition of MCAK to the ATP containing buffer. The concentration of MCAK or MCAK mutant used was 3 µM for ATPase assays in solution and 0.1 µM for assays in the presence of unpolymerized tubulin or microtubules. ATPase rates in solution were measured by monitoring the production of ADP using HPLC to separate ADP from ATP as described in [8,18]. ATPase rates in the presence of unpolymerized tubulin or microtubules were measured by linking the production of ADP to the oxidation of NADH as described in [31]. Unpolymerized GDP-tubulin was added at a final concentration of 10 µM. Double-cycled, GMPCPP-stabilized microtubules [29] were added at a final concentration of 10 µM polymerized tubulin. The oxidation of NADH was monitored via fluorescence (*λ*_ex_ = 340 nm, *λ*_em_ = 460 nm). The change in fluorescence intensity per unit time was obtained by fitting the initial signal change to a linear function. The change in fluorescence was converted into change in concentration of ADP using a standard curve. The change in concentration of ADP per unit time was then divided by the concentration of MCAK to give the ATPase activity per second per motor domain.

### 4.5. Single molecule TIRF

Single-cycled, rhodamine-labelled, GMPCPP-stabilized microtubules were stuck onto the surface of flow chambers prepared as described in [15,29]. MCAK-GFP or GFP-labelled MCAK mutants at single-molecule concentrations (1–6 nM) were added to a microtubule containing flow cell in BRB20 pH 6.9, 75 mM KCl, 1 mM ATP, 0.05% Tween 20, 0.1 mg ml^−1^ BSA, 1% 2-mercaptoethanol, 40 mM glucose, 40 mg ml^−1^ glucose oxidase, 16 mg ml^−1^ catalase.

Images were recorded using a Zeiss Observer Z1 microscope equipped with a Zeiss Laser TIRF 3 module, QuantEM 512SC EMCDD camera (Photometrics) and 100× objective (Zeiss, alphaPlanApo/1.46NA oil). Experiments were performed at 25°C. Images of rhodamine-labelled microtubules were recorded using a lamp as the excitation source (Zeiss, HBO 100) and Zeiss filter set 20. TIRF data (measuring GFP fluorescence emission) were collected using a 488 nm laser as the light source (nominal power at the objective of 2 mW), directed through the Zeiss TIRF 3 module and using Zeiss filter set 38. For both rhodamine and GFP imaging, an exposure time of 100 ms was used.

Images were collected using the following protocol: (i) a single image of rhodamine-labelled microtubules, (ii) 200 images of MCAK-GFP with TIRF illumination and the camera ‘streaming’ with a frame rate of 7.4 Hz and (iii) a second single image of rhodamine-labelled microtubules (to check that neither significant depolymerization nor movement of the stage had occurred). For analysis, each frame of the MCAK-GFP dataset was colour-combined with the corresponding image of rhodamine-labelled microtubules in Fiji [30] to enable identification of on-microtubule events. Kymographs for individual microtubules were used to measure the duration of individual GFP localization events at the microtubule end and on the lattice, also in Fiji [30].

### 4.6. Dissociation of mantADP

MCAK or MCAK variants were preloaded with mantADP as described in [18,19]. The kinetics of dissociation of mantADP from MCAK were measured by rapidly mixing 1 : 1 v/v, using an SX20 stopped-flow fluorimeter (Applied Photophysics) with an excess of unlabelled ATP at 25°C. The fluorescence of the mant group was excited at 365 nm and the emitted fluorescence collected using a BP445/50 filter (Zeiss). These assays were carried out either in solution, in the presence of 10 µM unpolymerized GDP-tubulin or in the presence of 5.7 µM double-cycled GMPCPP microtubules. The concentration of microtubules was chosen such that an equivalent number of microtubule ends were present in these assays as in the microtubule-stimulated ATPase assays. The same double-cycled GMPCPP-stabilized microtubules, which have an average length of 2.1 µm [8], were used in both. However, microtubules break when pushed through the stopped-flow, resulting in microtubules with an average length of 1.2 µm [8]. The resulting fluorescent transients were fitted using IGOR Pro (Wavemetrics, Lake Oswego, OR, USA) to a single or a double exponential function plus a line of constant negative slope to account for photobleaching of the mant group, as described in [19].

## Supplementary Material

Supplementary Information
