# Supplementary material for: The family-specific α4-helix of the kinesin-13, MCAK, is critical to microtubule end recognition
Source: Open Biol. 2016 Oct 12;6(10):160223. doi: 10.1098/rsob.160223 (PMC5090061; doi:10.1098/rsob.160223)
Supplement: Supplementary Information [file rsob160223supp1.pdf]

Supplementary Information (Open Biology)

The family specific α4 helix of the kinesin-13, MCAK, is critical to microtubule end recognition.

Jennifer T. Patel[1,2], Hannah R. Belsham[1,2], Alexandra J. Rathbone[2], Bill Wickstead[2], Christopher Gell[2] and Claire T. Friel[2*]

[1]These authors contributed equally to this work.

[2]School of Life Sciences
University of Nottingham
Medical School, QMC
Nottingham
NG7 2UH
UK

*corresponding author

1. Atypical ATP turnover cycle of MCAK

The kinesin-13, MCAK, has an atypical ATP turnover cycle, characterized by ATP cleavage (Step 2, k_2 (Supplementary Figure S1A) being rate limiting in solution rather than the more common rate-limiting step of ADP dissociation (Step 4, k_4 (Supplementary Figure S1A)[1]. The ATPase rate of WT-MCAK in solution (absence of tubulin or microtubules) is ~0.002 s^{-1}. This rate is set by the rate-limiting step which for MCAK in solution is ATP cleavage (Part A, Step 2, k_2). Tubulin in any form activates the ATP cleavage step such that it is no longer rate limiting. The ATPase rate in the presence of unpolymerized tubulin is ~0.15 s^{-1}. This is set by the rate of dissociation of ADP (Part A, Step 4, k_4). ADP dissociation is not accelerated by unpolymerized tubulin or the microtubule lattice but proceeds with the same rate constant observed in the absence of tubulin. The microtubule end-stimulated ATPase rate is ~3 s^{-1}. This is set by the rate of ADP dissociation (Part A, Step 4, k_4), which is accelerated more than 20-fold by the microtubule end.

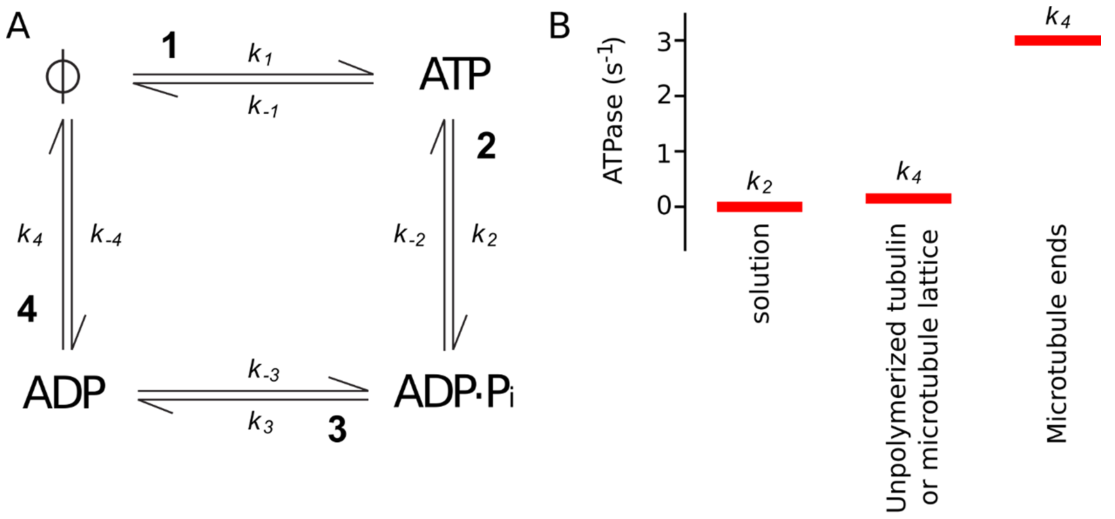

Supplementary Figure S1: A) Minimal ATP turnover cycle for an ATP hydrolysing enzyme. The cycle has 4 steps: 1) ATP binding to the nucleotide free (ϕ) binding site, 2) ATP cleavage to produce ADP and inorganic phosphate (P_i), 3) dissociation of inorganic phosphate (P_i), 4) dissociation of ADP. The kinetics of each step is represented by a forward and backward rate constant (k_x and k_{-x}). B) Comparison of the ATPase activity of WT-MCAK in solution, activated by unpolymerized tubulin or the microtubule lattice and activated by microtubule ends. The rate limiting step in each condition is shown (labelled according to the cycle shown in A) above the red bar which indicates the ATPase rate in each condition.

2. Selection of mutants in this study

The residues focused on in this study were chosen as they showed a large decrease in microtubule depolymerization activity in a screen of 23 point mutants at 19 locations in the MCAK motor domain. The positions of the mutants were chosen from a comparative protein sequence alignment of the motor domains of the Kinesin-1 and Kinesin-13 families (Supplementary Figure S2), we selected residues which discriminate for the Kinesin-13 family (*i.e.* highly conserved within Kinesin-13s, but dissimilar between the Kinesin-1 and Kinesin-13 families). Residues were changed either to alanine or, where a consensus existed, to the residue at the same position in the Kinesin-1 family. Measuring the depolymerization activity, by light scattering, showed 11 mutations which decreased depolymerization activity by more than 50% relative to WT-MCAK (Supplementary Figure S3).

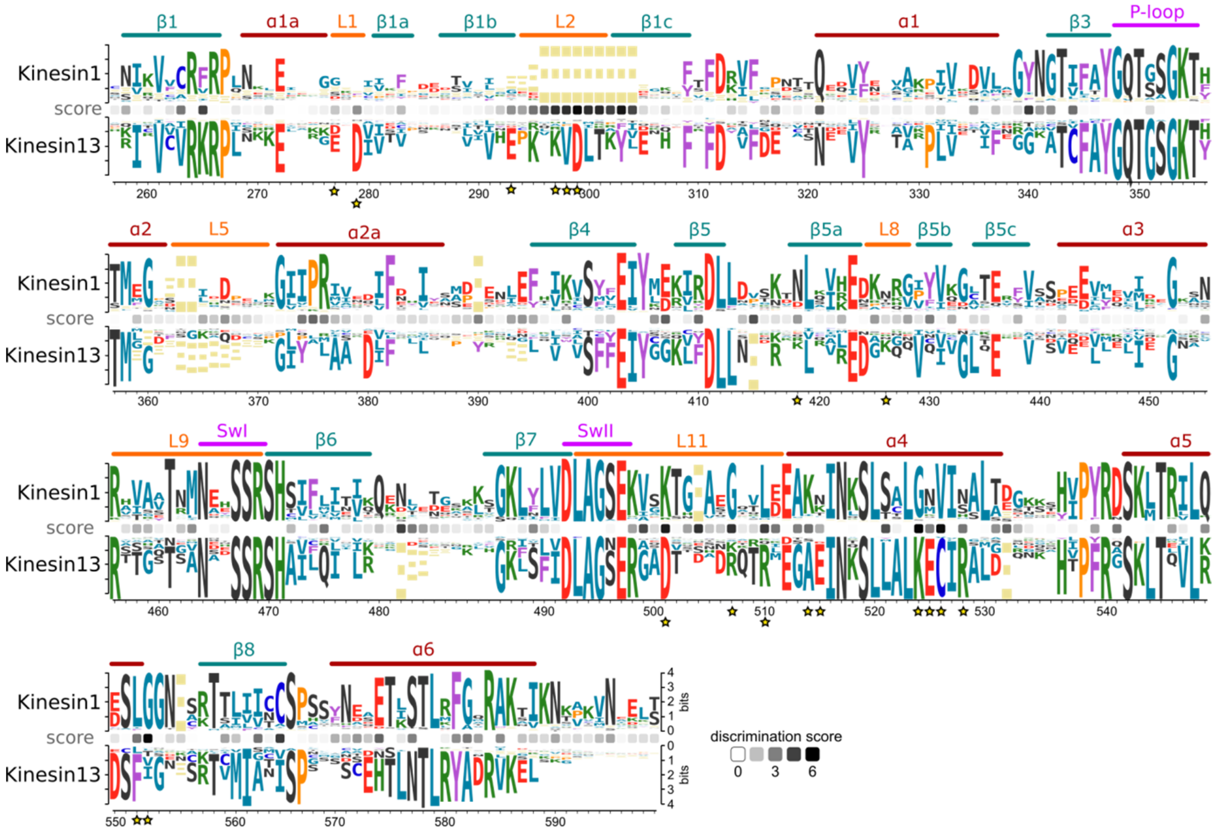

Supplementary Figure S2: Comparison of the motor domains of Kinesin-1 and -13 family members. The alignments of kinesins within each family from 45 diverse eukaryotes [2] are displayed as logos, in which the height of the residue represents the degree of conservation at that position (relative entropy[3]). Sequence differences between the Kinesin-1 and Kinesin-13 families are shown by the "discrimination score", which is the difference between the mean of the BLOSUM62 substitution matrix scores[4] when comparing all sequences within each family and the mean scores comparing all sequences between the families (darker = higher score). Residues are coloured according to biochemical grouping and positions numbered according to the HsMCAK sequence. Secondary structure elements from murine MCAK (PDB: 1V8K [5]) are shown above logos. The residues mutated are marked by yellow stars.

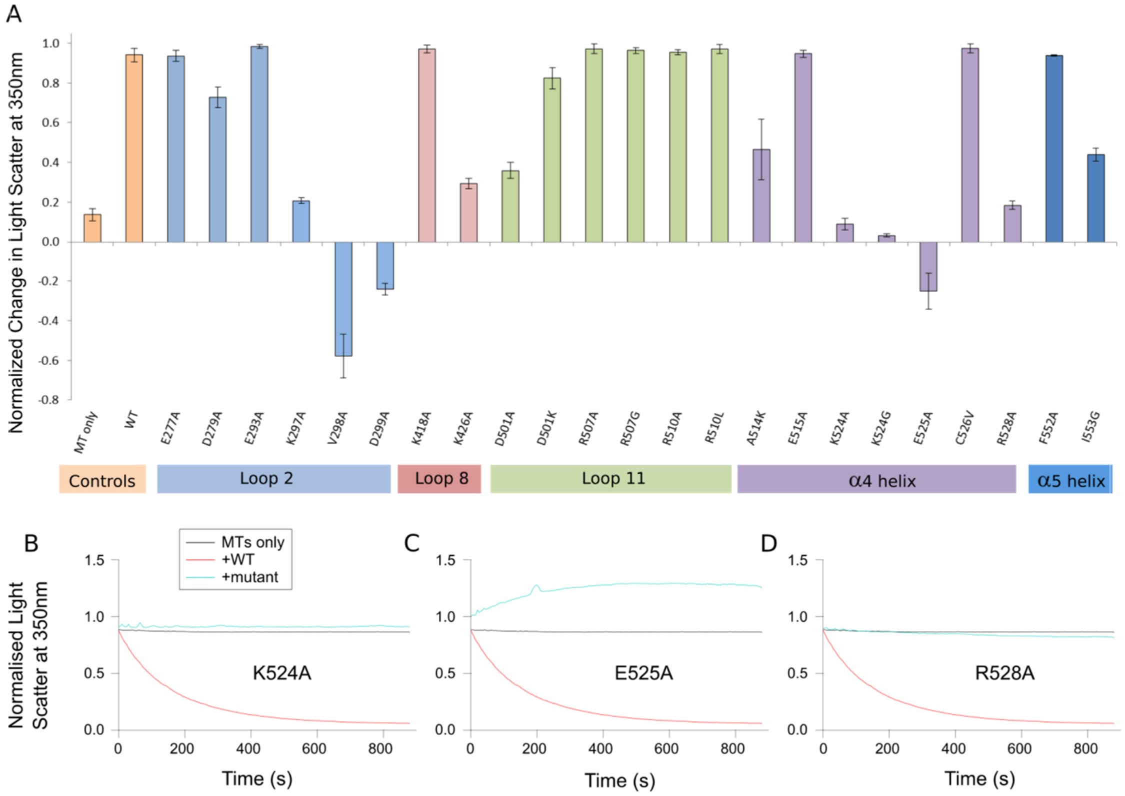

Supplementary Figure S3: A screen of the MCAK motor domain highlights point mutations which reduce microtubule depolymerization activity.

A) Depolymerization activity of MCAK point mutants monitored by light scattering. A large change in light scatter indicates microtubule depolymerization. Low signal change indicates mutants with impaired depolymerization activity. Negative values are likely due to mutants with impaired depolymerization activity which cross-link microtubules. B-D) Normalized light scatter signal with time upon adding WT-MCAK or MCAK mutants to microtubules. The traces for MCAK mutants are in blue: B) K524A, C) E525A and D) R528A. The traces for WT-MCAK are in red and microtubules alone in black. The increase in light scattering seen for E525A (also observed for V298A and D299A) is likely due to crosslinking of microtubules. MCAK is known to have the ability to crosslink microtubules and/or tubulin proto-filaments under certain conditions[1, 6].

3. Additional plots and data fitting

Supplementary Figures S4 and S5 show fits from which some of the parameters listed in Table 1 and in Supplementary Table S1 are determined.

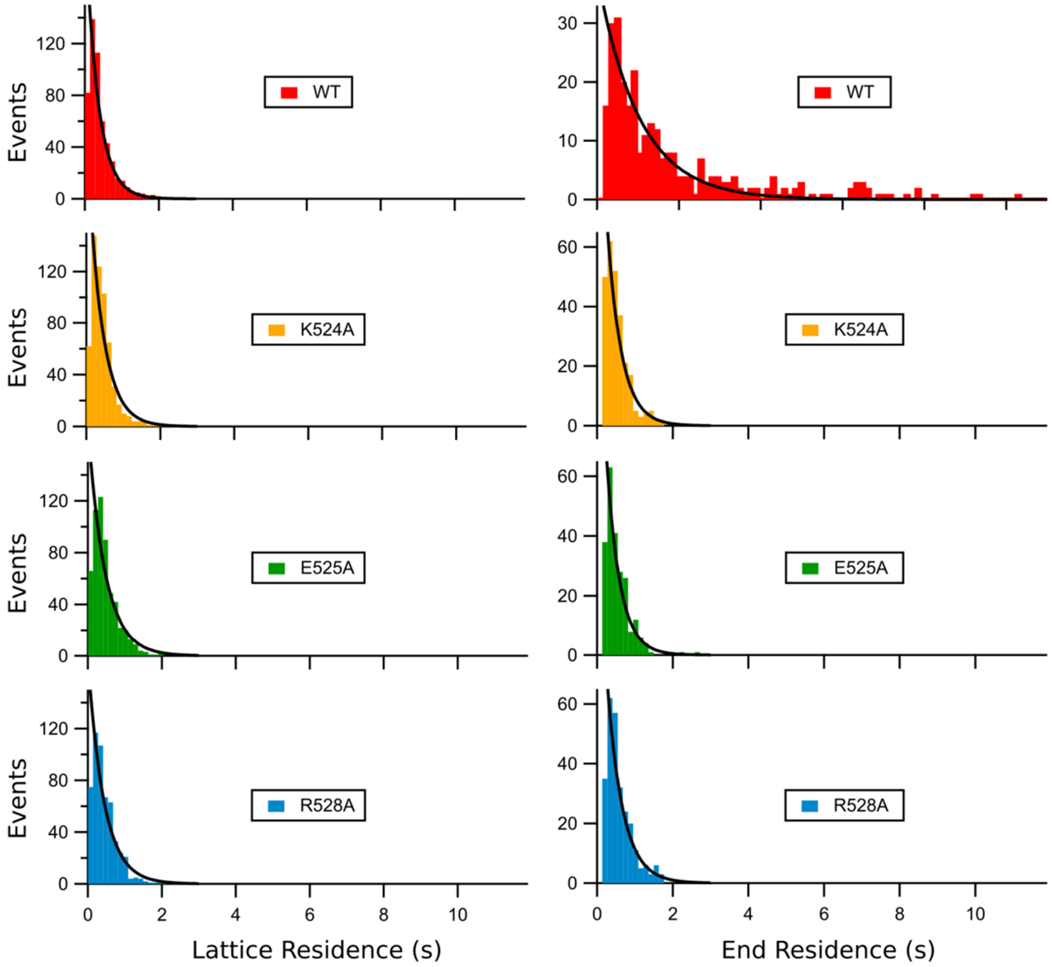

Supplementary Figure S4: Histograms showing the number of microtubule lattice and microtubule end interaction events of a particular residence time for WT-MCAK, K524A, E525A and R528A. The back lines are the fits of these data to a single exponential function. The microtubule lattice and microtubule end dissociation rate constants obtained from these fits are listed in Supplementary Table S1.

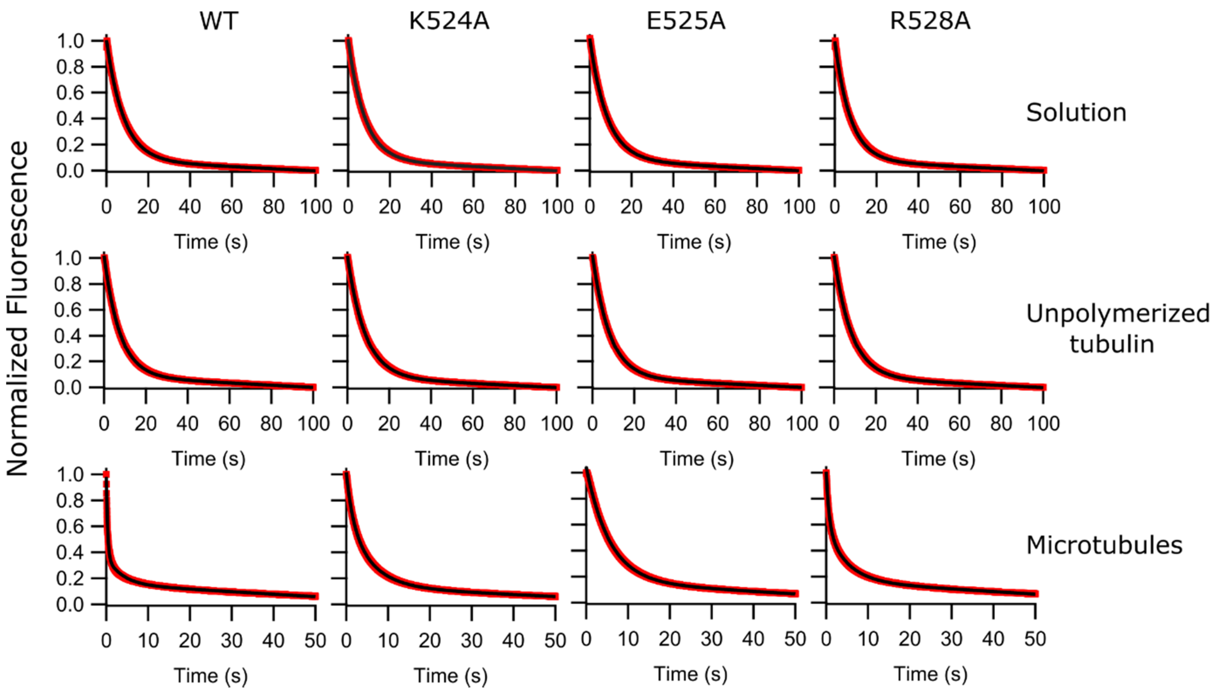

Supplementary Figure S5: Fluorescence transients (red) resulting from the dissociation of mantADP from the nucleotide binding site of WT-MCAK and the mutants K524A, E525A and R528A. Upper panel: mantADP dissociation in solution (absence of tubulin or microtubules). Middle panel: mantADP dissociation in the presence of unpolymerized tubulin. Lower panel: mantADP dissociation in the presence of microtubules. The fit to each transient of either a single or double exponential function plus a baseline of constant negative slope to account for photobleaching of the mant-labeled nucleotide is shown in black. All transients in the upper and middle panels are fit to a single exponential. All transients in the lower panel are fit to a double exponential, except E525A which fits well to a single exponential. The rate constants for ADP dissociation obtained from these fits are listed in Table 1.

The proportion of the total amplitude attributed to the first and second phases, respectively, of the double exponential fits are: WT 0.75 ± 0.03 and 0.25 ± 0.03 (mean ± SEM, n= 4), K524A 0.50 ± 0.07 and 0.50 ± 0.07 (mean ± SEM, n= 3) and R528A 0.42 ± 0.03 and 0.58 ± 0.03 (mean ± SEM, n= 4). The relative amplitudes for WT-MCAK are consistent with previous data[1].

Supplementary Table S1: Values for the microtubule lattice-residence times, lattice and end dissociation constants and microtubule association constants for WT-MCAK and the mutants K524A, E525A and R528A.
The microtubule-lattice and microtubule-end dissociation constants (k_{off}) were determined from fitting the histograms of lattice- and end-residence times to a single exponential function (Supplementary Figure S4). The association constants (k_{on}) were calculated as described in Helenius et al [7].

MCAK Variants	WT	K524A	E525A	R528A
		**(s)** (mean ± SEM)		
**Lattice residence time**	0.48 ± 0.02 (n = 526)	0.52 ± 0.03 (n = 587)	0.53 ± 0.02 (n = 559)	0.51 ± 0.02 (n = 527)
		**(s^{-1})** (mean ± SD)		
**k_{off}** (MT lattice)	2.90 ± 0.16	2.54 ± 0.22	2.81 ± 0.24	2.29 ± 0.18
**k_{off}** (MT end)	0.98 ± 0.06	2.66 ± 0.23	2.85 ± 0.17	2.41 ± 0.21
		**(s^{-1} nM^{-1} μm^{-1})** (mean ± SD)		
**k_{on}** (MT)	0.59 ± 0.28 (n = 3)	0.56 ± 0.04 (n = 3)	0.51 ± 0.14 (n = 3)	0.52 ± 0.30 (n = 3)

Supplementary References:

1. Friel, C.T. and Howard, J., (2011) *EMBO J*. **30**: p. 3928-39.
2. Wickstead, B., et al., (2010) *PLoS One*. **5**: p. e15020.
3. Schneider, T.D. and Stephens, R.M., (1990) *Nucleic Acids Res*. **18**: p. 6097-100.
4. Henikoff, S. and Henikoff, J.G., (1992) *Proc Natl Acad Sci U S A*. **89**: p. 10915-9.
5. Ogawa, T., et al., (2004) *Cell*. **116**: p. 591-602.
6. Tan, D., et al., (2008) *Structure*. **16**: p. 1732-9.
7. Helenius, J., et al., (2006) *Nature*. **441**: p. 115-9.
